# Association Between COVID-19 and Myocarditis Using Hospital-Based Administrative Data — United States, March 2020–January 2021

**DOI:** 10.15585/mmwr.mm7035e5

**Published:** 2021-09-03

**Authors:** Tegan K. Boehmer, Lyudmyla Kompaniyets, Amy M. Lavery, Joy Hsu, Jean Y. Ko, Hussain Yusuf, Sebastian D. Romano, Adi V. Gundlapalli, Matthew E. Oster, Aaron M. Harris

**Affiliations:** ^1^CDC COVID-19 Response Team; ^2^Emory University School of Medicine, Atlanta, Georgia; ^3^Children's Healthcare of Atlanta, Atlanta, Georgia.

Viral infections are a common cause of myocarditis, an inflammation of the heart muscle (myocardium) that can result in hospitalization, heart failure, and sudden death (*1*). Emerging data suggest an association between COVID-19 and myocarditis (*2*–*5*). CDC assessed this association using a large, U.S. hospital-based administrative database of health care encounters from >900 hospitals. Myocarditis inpatient encounters were 42.3% higher in 2020 than in 2019. During March 2020–January 2021, the period that coincided with the COVID-19 pandemic, the risk for myocarditis was 0.146% among patients diagnosed with COVID-19 during an inpatient or hospital-based outpatient encounter and 0.009% among patients who were not diagnosed with COVID-19. After adjusting for patient and hospital characteristics, patients with COVID-19 during March 2020–January 2021 had, on average, 15.7 times the risk for myocarditis compared with those without COVID-19 (95% confidence interval [CI] = 14.1–17.2); by age, risk ratios ranged from approximately 7.0 for patients aged 16–39 years to >30.0 for patients aged <16 years or ≥75 years. Overall, myocarditis was uncommon among persons with and without COVID-19; however, COVID-19 was significantly associated with an increased risk for myocarditis, with risk varying by age group. These findings underscore the importance of implementing evidence-based COVID-19 prevention strategies, including vaccination, to reduce the public health impact of COVID-19 and its associated complications.

Data for this study were obtained from the Premier Healthcare Database Special COVID-19 Release (PHD-SR), a large hospital-based administrative database.^†^ The monthly number of myocarditis^§^ and COVID-19^¶^ inpatient encounters was assessed before and during the COVID-19 pandemic, from January 2019 through May 2021.

A patient-level cohort was created to assess the association between COVID-19 and myocarditis. The cohort included all patients with at least one inpatient or hospital-based outpatient encounter with discharge during March 2020–January 2021. To minimize potential bias from vaccine-associated myocarditis (*6*), 277,892 patients with a COVID-19 vaccination record in PHD-SR during December 2020–February 2021 were excluded. In addition, 37,896 patients for whom information on sex was missing were excluded. Patients with COVID-19 were defined as those who had their first inpatient or outpatient encounter with a COVID-19 *International Classification of Diseases, Tenth Revision, Clinical Modification* (ICD-10-CM) code during March 2020–January 2021. Patients with myocarditis were defined as those who had their first of at least one inpatient encounter, at least two outpatient encounters, or at least one outpatient encounter with a relevant specialist** with a myocarditis ICD-10-CM code during March 2020–February 2021.^††^ Among patients with COVID-19, the first myocarditis encounter could have occurred during or after the first COVID-19 health care encounter.

The risk for myocarditis was defined as the percentage of patients with myocarditis and was calculated among patients with and without COVID-19, overall and by sex (male or female) and age group (<16, 16–24, 25–39, 40–49, 50–64, 65–74, and ≥75 years). The percentage of myocarditis patients with a history of COVID-19 was calculated for each age group.

Associations between COVID-19 and myocarditis were estimated using a multiple logit model with the following covariates: three-way interaction between COVID-19, sex, and age group, including lower-order interactions and main effects; race/ethnicity; payer type; hospital U.S. Census region; and hospital urbanicity. Adjusted risk differences (aRDs, measure of absolute risk) were calculated as the difference between 1) the adjusted predicted risk for myocarditis (outcome) among patients with COVID-19 (exposed group) and 2) adjusted predicted risk for myocarditis among patients without COVID-19 (unexposed group); adjusted risk ratios (aRRs, measure of relative risk) were calculated as the ratio of the adjusted predicted risk among exposed to the adjusted predicted risk among unexposed^§§^ (*7*,*8*). All models used standard errors clustered on a unique hospital identifier. R (version 4.0.2; R Foundation) and Stata (version 15.1; StataCorp) were used to conduct all analyses. This activity was reviewed by CDC and was conducted consistent with applicable federal law and CDC policy.^¶¶^

During 2020, the number of myocarditis inpatient encounters (4,560) was 42.3% higher than that during 2019 (3,205). Peaks in myocarditis inpatient encounters during April–May 2020 and November 2020–January 2021 generally aligned with peaks in COVID-19 inpatient encounters (Figure 1). 

**FIGURE 1:**
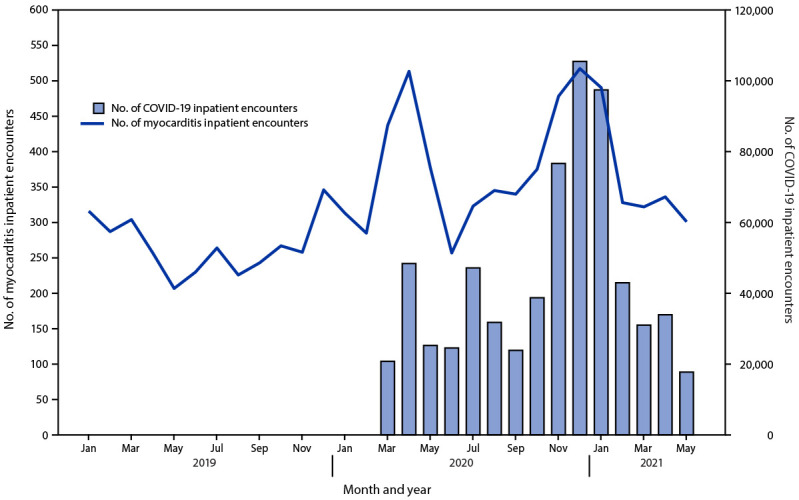
Number of myocarditis and COVID-19 inpatient encounters, by month* — Premier Healthcare Database Special COVID-19 Release, United States, January 2019–May 2021 * Data from recent months might be incomplete.

Within the cohort of 36,005,294 patients, 1,452,773 (4.0%) received a diagnosis of COVID-19 during March 2020–January 2021, and 5,069 (0.01%) received a diagnosis of myocarditis during March 2020–February 2021. Overall, 4,339 (85.6%) patients with myocarditis were identified by an inpatient encounter. Patients with myocarditis were slightly older than patients without myocarditis (median age = 54 years versus 50 years) and were more commonly male (59.3% versus 41.7%) (Supplementary Table, https://stacks.cdc.gov/view/cdc/109261).

Among patients with myocarditis, 2,116 (41.7%) had a history of COVID-19; this percentage was similar among males (42.4%) and females (40.9%) and differed by age group, with the lowest percentages among persons aged 16–24 years (23.7%) and 25–39 years (24.1%) and the highest among adults aged ≥75 years (64.6%) (Table). Among the 2,116 patients with COVID-19 and myocarditis, 1,895 (89.6%) received a diagnosis of COVID-19 and myocarditis during the same month; the remaining patients received a myocarditis diagnosis 1 month (139; 6.6%) or ≥2 months (82; 3.9%) after their COVID-19 diagnosis.

**TABLE:** Frequency of and risk for myocarditis among patients with and without COVID-19 and adjusted* myocarditis risk differences and risk ratios comparing patients with and without COVID-19 — Premier Healthcare Database Special COVID-19 Release, United States, March 2020–January 2021

Characteristic	No. of patients with COVID-19	No. of patients without COVID-19	No. of patients with myocarditis	Myocarditis among patients with COVID-19		Myocarditis among patients without COVID-19		Adjusted myocarditis risk difference (95% CI)	Adjusted myocarditis risk ratio (95% CI)
				No. (% of patients with myocarditis)	Risk, %	No. (% of patients with myocarditis)	Risk, %		
**Overall**	1,452,773	34,552,521	5,069	2,116 (41.7)	0.146	2,953 (58.3)	0.009	0.126 (0.112–0.140)	15.7 (14.1–17.2)
**Sex**									
Male	680,722	14,339,356	3,008	1,274 (42.4)	0.187	1,734 (57.6)	0.012	0.165 (0.146–0.183)	13.8 (12.3–15.3)
Female	772,051	20,213,165	2,061	842 (40.9)	0.109	1,219 (59.1)	0.006	0.100 (0.087–0.113)	17.8 (15.6–20.0)
**Age group, yrs**									
<16	64,898	3,670,762	218	86 (39.4)	0.133	132 (60.6)	0.004	0.122 (0.065–0.179)	36.8 (25.0–48.6)
16–24	123,865	3,067,575	511	121 (23.7)	0.098	390 (76.3)	0.013	0.088 (0.061–0.115)	7.4 (5.5–9.2)
25–39	268,549	6,246,568	862	208 (24.1)	0.077	654 (75.9)	0.010	0.067 (0.052–0.081)	6.7 (5.5–8.0)
40–49	198,561	4,147,909	620	213 (34.4)	0.107	407 (65.6)	0.010	0.093 (0.078–0.109)	10.0 (8.1–11.9)
50–64	356,697	7,965,264	1,226	553 (45.1)	0.155	673 (54.9)	0.008	0.137 (0.121–0.154)	17.0 (14.7–19.3)
65–74	214,331	5,318,474	801	398 (49.7)	0.186	403 (50.3)	0.008	0.160 (0.135–0.184)	23.0 (19.4–26.7)
≥75	225,872	4,135,969	831	537 (64.6)	0.238	294 (35.4)	0.007	0.208 (0.179–0.237)	31.6 (25.9–37.2)

**Abbreviation:** CI = confidence interval.

* Adjusted risk differences and risk ratios for myocarditis during or after COVID-19 (reference group: no COVID-19), obtained from a single logit model with the following covariates: a three-way interaction between presence of COVID-19, sex, and age group, including lower-order interactions and main effects; race/ethnicity; payer type; hospital U.S. Census region; and hospital urbanicity.

During March 2020–January 2021, the risk for myocarditis was 0.146% among patients with COVID-19 and 0.009% among patients without COVID-19. Among patients with COVID-19, the risk for myocarditis was higher among males (0.187%) than among females (0.109%) and was highest among adults aged ≥75 years (0.238%), 65–74 years (0.186%), and 50–64 years (0.155%) and among children aged <16 years (0.133%). 

In adjusted analyses, patients with COVID-19 had, on average, 15.7 (95% CI = 14.1–17.2) times the risk for myocarditis compared with patients without COVID-19; however, because of the low risk for myocarditis in both groups, the aRD between patients with and without COVID-19 was small (aRD = 0.126%; 95% CI = 0.112%–0.140%) (Table) (Figure 2). The aRRs of myocarditis was higher among females (17.8; 95% CI = 15.6–20.0) than among males (13.8; 95% CI = 12.3–15.3), whereas the aRD was higher among males (0.165%; 95% CI = 0.146%–0.183%) than among females (0.100%; 95% CI = 0.087%–0.113%). The aRR and aRD were lowest for patients aged 25–39 years and were higher among younger and older age groups. The aRRs ranged from approximately 7.0 for patients aged 16–24 and 25–39 years to >30.0 for patients aged <16 years and ≥75 years.

**FIGURE 2:**
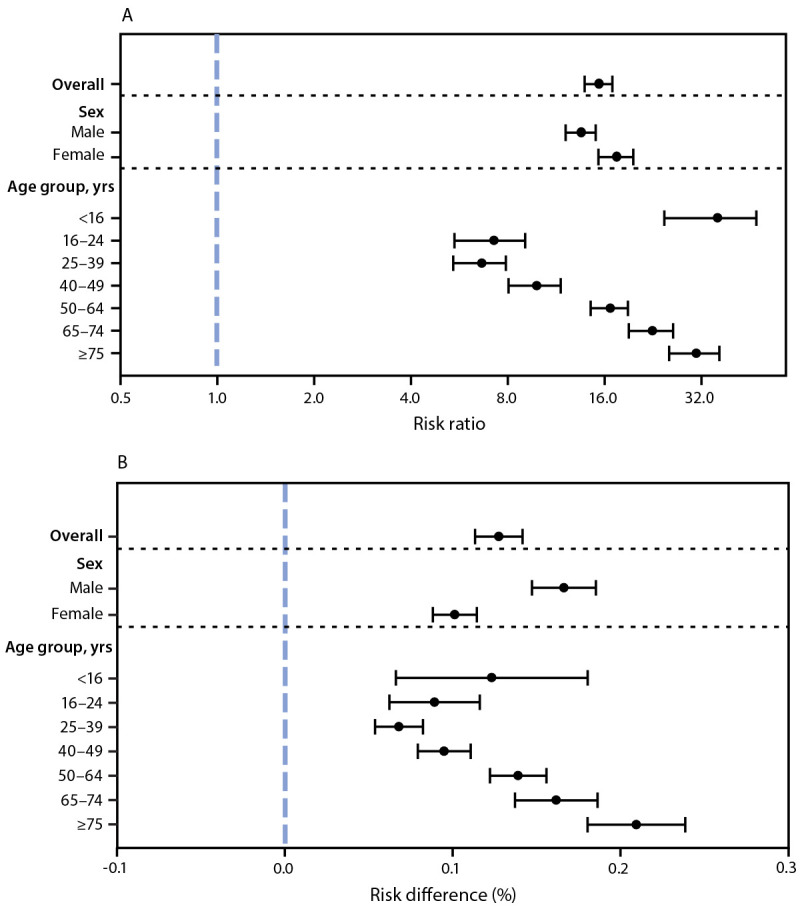
Adjusted risk ratio (A) and adjusted risk difference (B) of myocarditis comparing patients with and without COVID-19,* overall and by sex and age group — Premier Healthcare Database Special COVID-19 Release, United States, March 2020–January 2021 * The panels show adjusted risk ratios (A) and adjusted risk differences (B) of myocarditis comparing patients with COVID-19 to patients without COVID-19 (reference), obtained from a single logit model with the following covariates: a three-way interaction between presence of COVID-19, sex, and age group, including lower-order interactions and main effects; race/ethnicity; payer type; hospital U.S. Census region; and hospital urbanicity. 95% confidence intervals indicated by error bars.

## Discussion

In this study, the occurrence of myocarditis inpatient encounters was 42% higher in 2020 than in 2019. The risk for myocarditis among patients with COVID-19 during March 2020–January 2021 was nearly 16 times as high as the risk among patients without COVID-19, with the association between COVID-19 and myocarditis being most pronounced among children and older adults. Further, in this cohort, approximately 40% of patients with myocarditis had a history of COVID-19.

These findings suggest an association between COVID-19 and myocarditis, although causality cannot be inferred from observational data, and are consistent with those from previous studies (*2*–*5*). Before this report, the two largest known studies, in the United States and in Israel, also found that COVID-19 was strongly associated with myocarditis (U.S. study: odds ratio = 8.17, 95% CI = 3.58–18.62; Israel study: risk ratio = 18.28, 95% CI = 3.95–25.12) (*3*,*4*).

In this study, the association between COVID-19 and myocarditis was lowest for persons aged 25–39 years and higher among younger (<16 years) and older (≥50 years) age groups, a pattern that has not been previously described in age-stratified analyses and that warrants further investigation. This finding might be partially explained by age-related differences in COVID-19 case ascertainment, because younger adults with less severe disease might be less likely than older adults to have a health care encounter with a COVID-19 diagnosis captured within PHD-SR. This age-related differential misclassification (underascertainment) of COVID-19 status might bias risk differences and risk ratios toward the null more for younger adults and could partially explain the observed age-related association.

The risk difference for myocarditis between persons with and without COVID-19 was higher among males than among females, consistent with some earlier studies (*2*,*5*). The finding of a higher risk ratio among females than among males is novel. However, it likely reflects the low risk for myocarditis among female patients without COVID-19 (*5*).

Although the exact mechanism of SARS-CoV-2 infection possibly leading to myocarditis is unknown, the pathophysiology is likely similar to that of other viruses (*1*). Among persons with COVID-19 and myocarditis, some myocarditis diagnoses might represent cases of multisystem inflammatory syndrome (MIS), particularly among children aged <16 years (*9*). Further study is warranted to understand how the clinical course of myocarditis among patients with COVID-19 might differ by presence or absence of MIS (*10*).

Since the introduction of mRNA COVID-19 vaccines in the United States in December 2020, an elevated risk for myocarditis among mRNA COVID-19 vaccine recipients has been observed, particularly among males aged 12–29 years, with 39–47 expected cases of myocarditis, pericarditis, and myopericarditis per million second mRNA COVID-19 vaccine doses administered (*6*). A recent study from Israel reported that mRNA COVID-19 vaccination was associated with an elevated risk for myocarditis (risk ratio = 3.24; 95% CI = 1.55–12.44); in the same study, a separate analysis showed that SARS-CoV-2 infection was a strong risk factor for myocarditis (risk ratio = 18.28, 95% CI = 3.95–25.12) (*4*). On June 23, 2021, the Advisory Committee on Immunization Practices concluded that the benefits of COVID-19 vaccination clearly outweighed the risks for myocarditis after vaccination (*6*). The present study supports this recommendation by providing evidence of an elevated risk for myocarditis among persons of all ages with diagnosed COVID-19.

The findings in this study are subject to at least six limitations. First, the risk estimates from this study reflect the risk for myocarditis among persons who received a diagnosis of COVID-19 during an outpatient or inpatient health care encounter and do not reflect the risk among all persons who had COVID-19. Second, misclassification of COVID-19 and myocarditis is possible because conditions were determined by ICD-10-CM codes, which were not confirmed by clinical data (e.g., laboratory tests or cardiac imaging) and could be improperly coded or coded with a related condition (e.g., pericarditis). Third, encounters for COVID-19, myocarditis, and COVID-19 vaccination occurring outside of hospital systems that contribute to PHD-SR are not included within this data set. Fourth, underlying medical conditions and alternative etiologies for myocarditis (e.g., autoimmune disease) were not ascertained or excluded. Fifth, the obtained measures of association could be biased because of the choice of the comparison group (all patients without COVID-19) and if physicians were more likely to suspect or diagnose myocarditis among patients with COVID-19. Finally, the findings represent a convenience sample of patients from hospitals reporting to PHD-SR and might not be generalizable to the U.S. population.

Myocarditis is uncommon among patients with and without COVID-19; however, COVID-19 is a strong and significant risk factor for myocarditis, with risk varying by age group. The findings in this report underscore the importance of implementing evidence-based COVID-19 prevention strategies, including vaccination, to reduce the public health impact of COVID-19 and its associated complications.

**Summary**

**What is already known about this topic?**

Viral infections are a common cause of myocarditis. Some studies have indicated an association between COVID-19 and myocarditis.

**What is added by this report?**

During March 2020–January 2021, patients with COVID-19 had nearly 16 times the risk for myocarditis compared with patients who did not have COVID-19, and risk varied by sex and age.

**What are the implications for public health practice?**

These findings underscore the importance of implementing evidence-based COVID-19 prevention strategies, including vaccination, to reduce the public health impact of COVID-19 and its associated complications.

## Footnotes

* These authors contributed equally to this report.

† PHD-SR, formerly known as the PHD COVID-19 Database, is a large U.S. hospital-based all-payer database that includes inpatient and hospital-based outpatient (e.g., emergency department or clinic) health care encounters from >900 geographically diverse, nonprofit, nongovernmental, community, and teaching hospitals and health systems from rural and urban areas. PHD-SR represents approximately 20% of inpatient admissions in the United States. Updated PHD-SR data are released every 2 weeks; release date August 4, 2021, access date August 4, 2021. http://offers.premierinc.com/rs/381-NBB-525/images/PHD_COVID-19_White_Paper.pdf

§ Myocarditis was identified by the following *International Classification of Diseases, Tenth Revision, Clinical Modification* (ICD-10-CM) codes: B33.20, B33.22, B33.24, I40.0, I40.1, I40.8, I40.9, or I51.4.

¶ COVID-19 was identified by ICD-10-CM code B97.29 (other coronavirus as the cause of diseases classified elsewhere) during March–April 2020 or ICD-10-CM code U07.1 (COVID-19, virus identified [laboratory-confirmed]) during or after April 2020. ICD-10-CM code B97.29 was recommended before the April 1, 2020, release of ICD-10-CM code U07.1. https://www.cdc.gov/nchs/data/icd/Announcement-New-ICD-code-for-coronavirus-3-18-2020.pdf

** Attending physician with one of the following specializations: cardiac electrophysiology, cardiovascular diseases, cardiovascular surgery, infectious diseases, or rheumatology.

†† Myocarditis was assessed using all encounters for each patient from January 2019 to February 2021. Patients were defined as having new myocarditis during the study period if their first myocarditis encounter in PHD-SR occurred during or after March 2020. Myocarditis was assessed through February 2021 to allow for myocarditis diagnoses to occur after COVID-19 diagnoses in January 2021.

§§ First, a multiple logit model was performed with the following covariates: three-way interaction between COVID-19, sex, and age group, including lower-order interactions and main effects; race/ethnicity; payer type; hospital U.S. Census region; and hospital urbanicity. Then the following average predicted probabilities (predictive margins) were estimated: 1) P1: the average predicted probability of myocarditis with COVID-19 set to be present and all other covariates set to their original values, and 2) P0: the average predicted probability of myocarditis with COVID-19 set to be absent and all other covariates set to their original values. aRD represents the difference in predicted probabilities (P1 minus P0); aRR represents the ratio of the predicted probabilities (P1 divided by P0). aRRs and aRDs were obtained for the full sample (where all covariates were set at their original values), for each age group (where age was set at the specific category of interest and other covariates were set at their original values), and for each sex (where sex was set at the specific category of interest and other covariates were set at their original values).

¶¶ 45 C.F.R. part 46, 21 C.F.R. part 56; 42 U.S.C. Sect. 241(d); 5 U.S.C. Sect. 552a; 44 U.S.C. Sect. Sect. 3501 et seq.

## References

[1] Pollack A, Kontorovich AR, Fuster V, Dec GW. Viral myocarditis—diagnosis, treatment options, and current controversies. Nat Rev Cardiol 2015;12:670–80. 10.1038/nrcardio.2015.108 26194549

[2] Rathore SS, Rojas GA, Sondhi M, Myocarditis associated with Covid-19 disease: a systematic review of published case reports and case series. Int J Clin Pract 2021;e14470. Epub July 7, 2021. 34235815 10.1111/ijcp.14470

[3] Murk W, Gierada M, Fralick M, Weckstein A, Klesh R, Rassen JA. Diagnosis-wide analysis of COVID-19 complications: an exposure-crossover study. CMAJ 2021;193:E10–8. 10.1503/cmaj.201686 33293424 PMC7774475

[4] Barda N, Dagan N, Ben-Shlomo Y, Safety of the BNT162b2 mRNA COVID-19 vaccine in a nationwide setting. N Engl J Med 2021.Epub August 25, 2021. 10.1056/NEJMoa2110475 34432976 PMC8427535

[5] Daugherty SE, Guo Y, Heath K, Risk of clinical sequelae after the acute phase of SARS-CoV-2 infection: retrospective cohort study. BMJ 2021;373:n1098. 10.1136/bmj.n1098 34011492 PMC8132065

[6] Gargano JW, Wallace M, Hadler SC, Use of mRNA COVID-19 vaccine after reports of myocarditis among vaccine recipients: update from the Advisory Committee on Immunization Practices—United States, June 2021. MMWR Morb Mortal Wkly Rep 2021;70:977–82. 10.15585/mmwr.mm7027e2 34237049 PMC8312754

[7] Kleinman LC, Norton EC. What’s the risk? A simple approach for estimating adjusted risk measures from nonlinear models including logistic regression. Health Serv Res 2009;44:288–302. 10.1111/j.1475-6773.2008.00900.x 18793213 PMC2669627

[8] Norton EC, Miller MM, Kleinman LC. Computing adjusted risk ratios and risk differences in Stata. Stata J 2013;13:492–509. 10.1177/1536867X1301300304

[9] Most ZM, Hendren N, Drazner MH, Perl TM. Striking similarities of multisystem inflammatory syndrome in children and a myocarditis-like syndrome in adults. Circulation 2021;143:4–6. 10.1161/CIRCULATIONAHA.120.050166 32787714

[10] Vukomanovic VA, Krasic S, Prijic S, Differences between pediatric acute myocarditis related and unrelated to SARS-CoV-2. Pediatr Infect Dis J 2021;40:e173–8. 10.1097/INF.0000000000003094 33847291

